# Surveying prioritisation for emergency surgery - do the specialties involved agree?

**DOI:** 10.1186/s12893-026-03538-3

**Published:** 2026-01-28

**Authors:** Katja Junus, Teodor Svedung-Wettervik, Karl Stattin, Erik Osterman

**Affiliations:** 1 Department of Women’s and Children’s Health, Uppsala University, Uppsala, Sweden; 2 Department of Medical Sciences, Uppsala University, Uppsala, Sweden; 3 Department of Surgical Sciences, Uppsala University, Akademiska Sjukhuset, Uppsala, 751 85 Sweden; 4 Department of Obstetrics and Gynaecology, Uppsala University Hospital, Uppsala, Sweden; 5 Department of Neurosurgery, Uppsala University Hospital, Uppsala, Sweden; 6 Department of Anesthesiology and Intensive Care, Uppsala University Hospital, Uppsala, Sweden; 7 Department of Surgery and Urology, Uppsala University Hospital, Uppsala, Sweden

**Keywords:** Emergency surgery, Conflicts, Patient safety

## Abstract

**Background:**

Surgical resources are often shared across medical specialties, which can lead to conflicts over prioritisation during on-call hours. This study investigated factors contributing to inter-speciality conflict in the prioritisation of emergency surgical cases.

**Methods:**

A survey on views of surgical urgency and prioritisation of 22 hypothetical cases was distributed to Swedish physicians involved in emergency surgery via posters in operating departments and administrative areas, and by email. The Kruskal-Wallis test and Dunn’s post-hoc test were used to compare responses across specialties. Factors associated with daily conflicts were identified using logistic regression.

**Results:**

Of 233 respondents, 201 (90%) reported sharing resources with other specialties. General surgeons (45%), orthopaedic surgeons (15%), and anaesthetists (15%) were the most represented. Over half reported insufficient resources for emergency surgery. Sixty-nine per cent perceived that other specialties over-prioritised their own patients. Thirty-nine respondents (17%) experienced daily conflicts over case prioritisation. Minor differences were observed in how respondents perceived their own prioritisation, that of other specialties, and the prioritisation of hypothetical cases. Daily conflicts were associated with concerns about compromised patient safety (odds ratio [OR] 8.05 and 95% confidence interval [CI] 1.6-63.5 for agreeing vs. strongly disagreeing/disagreeing), whereas greater perceived resource availability was associated with fewer conflicts (OR 0.68, 95% CI 0.48–0.96).

**Conclusions:**

Perceived threats to patient safety and resource scarcity were associated with daily prioritisation conflicts. Although respondents frequently suspected over-prioritisation by other specialties, observed differences in prioritisation between the responsible specialty and others were small. Further research on shared prioritisation protocols and resource allocation, including international comparisons across health-care systems and cultures, is warranted.

**Supplementary Information:**

The online version contains supplementary material available at 10.1186/s12893-026-03538-3.

## Background

Surgical urgency is often divided into immediate, urgent, and semi-urgent priority, although classification systems vary – using colour codes, timestamps, or defined time windows. In Sweden, a widely used classification includes the categories immediate, within 2 h, within 6 h, within 24 h, over 24 h (semi-urgent), and elective procedures [1–3]. Time to surgery is typically determined by patient condition and the expected clinical progression; for example, a life-threatening haemorrhage requires immediate intervention, while a threatening bowel obstruction from a tumour may need surgery within a few days. The World Society of Emergency Surgery (WSES) has summarised urgency guidelines across specialties [4]. 

Surgical resources, e.g. theatres, surgical nurses, anaesthesia teams, etc., are often shared among specialties, which can lead to conflict - especially when patients have similar urgency levels or when there is disagreement about medical urgency. Whether the prioritisation is perceived as medically justified may depend on the physician’s understanding of other specialties’ diseases and priorities. However, the extent of such conflicts and their impact on the work environment remain insufficiently studied. It is also unclear whether greater cross-specialty understanding of prioritisation practices influences these dynamics.

The study aimed to investigate how surgical prioritisation varies across specialties and its relationship to perceived workplace conflict in the Swedish context. The hypothesis was that physicians have a limited understanding of other specialties’ prioritisation criteria, contributing to conflicts regarding resources.

## Methods

A cross-sectional survey was developed, consisting of 34 questions divided into five sections, each on its own page: respondent background; work environment; time-based prioritisation of 12 hypothetical emergency cases; prioritisation by ranking four cases scheduled for surgery within 6 h; and prioritisation by ranking six cases scheduled for surgery within 24 h. Physician background variables included sex, years in practice, speciality, and involvement in emergency surgery prioritisation and resource-sharing between specialties at the respondent’s hospital. The perceived impact of prioritisation on patient safety and conflict was assessed using Likert-scale items.

Hypothetical case scenarios were designed in consultation with an anaesthetist, three general surgeons, one obstetrician and one gynaecologist, an orthopaedic surgeon, a neurosurgeon, a paediatric surgeon and urologist and a vascular surgeon to reflect diverse prioritisation challenges in anaesthesiology, general surgery, obstetrics and gynaecology, neuro-, orthopaedic-, paediatric-, urologic-, and vascular surgery. The review resulted in rewording of some cases to become more urgent and controlling the medical accuracy of the text. An additional case concerning the placement of a subcutaneous vascular access device was included, as this procedure may be performed by anaesthetists, general-, paediatric- or vascular surgeons depending on the hospital. Multiple cases per speciality were included (Table 1), guided by the World Society of Emergency Surgery Timing in Acute Care Surgery classification system for surgical urgency to capture different urgency levels [4]. Respondents were first asked to assign a priority to 12 cases, choosing between immediate surgery or surgery within 2 h, 6 h, or 24 h. They were then presented with four cases already prioritised for surgery within 6 h and asked to rank them according to urgency (i.e. which case should be operated on first, second and so forth). Finally, respondents were asked to rank six cases prioritised for surgery within 24 h in the same way.

**Table 1:** Cases that respondents prioritised in the survey

Short name	Speciality	Case description
		Cases to be prioritised for immediate, < 2, <6 and < 24 h surgery
Surg App	General & Paediatric Surgery	Laparoscopic appendectomy. 13-year-old boy with appendicitis. CRP 50. Local peritonitis. No fever. Not septic.
Surg Perf	General	Laparotomy. 56year old man with diverticulitis. Computed tomography with free fluid and some gas. Septic with peritonitis.
Neur cSDH	Neurosurgery	Evacuation of chronic subdural hematoma. 78-year-old man, prostate cancer, hypertension, and alcohol abuse. Increasing hemiparesis, very weak against gravity. Computed tomography with an expansive chronic subdural hematoma.
Neur aSAH	Neurosurgery	External ventricular drainage. 54-year-old woman, smoker, hypertension. Subarachnoid haemorrhage from ACom aneurysm. Declining level of consciousness RLS1/GCS 15 to RLS3a / GCS 11. Progression of hydrocephalus on control Computed Tomography.
OB/GYN Bleeding	*Obstetrics and Gynaecology*	Removal of retained placenta. 32-year-old woman with postpartum haemorrhage. So far 1500 ml of bleeding.
OB/GYN Torsion	*Obstetrics and Gynaecology*	Laparoscopy. 17-year-old with suspected ovarian torsion. Severe pain. Otherwise well.
Ort HipFx	Orthopaedic	Sliding hip screw and plate. 85-year-old woman. A stroke 2 months ago. Pertrochanteric femur fracture after fall.
Ort ExFix	Orthopaedic	Debridement and external fixation. 25-year-old woman with open tibia fracture after motocross accident. Impaired circulation in the foot.
Uro Torsion	Urology & Paediatric surgery	Exploration of testicle. 8-year-old with suspected testicular torsion. Pain since 4 h. Otherwise well.
Uro JJ	Urology	Double J-stent. 55-year-old man with urinary tract stones. Fever. No sepsis.
Vasc CTEA	Vascular	Carotid endarterectomy. 76-year-old woman with a stroke 2 days ago. 90% stenosis of left carotid.
Vasc Ischemia	Vascular	Thromboembolectomy lower extremity. 60-year-old man with atrial fibrillation. Cold, pulseless leg with loss of sensation.
		**Cases awaiting surgery within 6 h that respondents ranked based on urgency**
Surg App2	General	Laparoscopic appendectomy. 17-year-old woman with appendicitis. Fever. Peritonitis.
OB/GYN Ect	*Obstetrics and Gynaecology*	Laparoscopy. 25-year-old woman with ectopic pregnancy, not ruptured. Stable blood pressure.
Ort Disl	Orthopaedic	Closed reduction under general anaesthesia. 24-year-old man with elbow dislocation.
Uro JJ2	Urology	Double J-stent. 55-year-old woman with suspected blocked pyelitis due to ureteral stone.
		**Cases awaiting surgery within 24 h that respondents ranked based on urgency**
Anest ScVP	Anaesthesia/Access surgery	Subcutaneous venous port. 18-year-old male. Leukaemia.
Surg Stoma	General	Placement of a transversostomy. 50-year-old man. Obstructing rectal cancer.
Neur cSDH	Neurosurgery	Evacuation of chronic subdural hematoma. 71-year-old woman. Moderate bilateral subdural hematomas. Head injury 6 weeks ago, mild headache and balance problems.
OB/GYN Abort	*Obstetrics and Gynaecology*	Surgical abortion. 39-year-old woman. Intrauterine pregnancy without bleeding.
Ort HipFx2	Orthopaedic	Hemiprosthesis. 85-year-old woman. Hip fracture.
Vasc Thromb	Vascular	Thrombolysis. 73-year-old man. Critical ischemia with pain and ulceration in the right leg.

*CRP *C-reactive Protein,* GCS *Glasgow Coma Scale,* RLS *Reaction Level Scale

Full details of the survey questions and response formats are provided in Appendix 1. The target population was physicians involved in emergency surgery prioritisation. The survey was evaluated in a convenience sample of ten emergency general surgeons and surgical residents who were deemed similar to the intended target population, before wider distribution. The survey was available in Swedish and English. No sensitive personal data was collected, and ethical approval was therefore not required under Swedish and European Union legislation. Participants were informed about data handling in accordance with the General Data Protection Regulation. The survey was built using REDCap, hosted at Uppsala University [5]. The survey was available from 13 May to 10 October 2025 at redcap.link/surgprio with all responses collected electronically.

Distribution occurred via posters in administrative areas and operating departments at Uppsala University Hospital, and via email invitations sent to researchers and physicians affiliated with the departments for the above specialties at Sweden’s seven medical schools. Respondents could refer colleagues by email, expanding the reach. In total, 856 individuals were invited by email. The link distributed in the email was personalised, but a generic link was also available for use when forwarding the invitation. To preserve participant anonymity and facilitate survey dissemination, no additional measures were taken to prevent multiple participation.

### Data Preparation

Educational level was grouped into three categories (registrar, consultant, other). Years of experience was categorised as < 5 years, 5–10 years, 10–15 years, 15–20 years, and > 20 years. Specialty was encoded based on the subspecialty most relevant to emergency surgery prioritisation; for example, a general surgeon with a urologic speciality was categorised as a urologic surgeon. Sharing resources with four or more other specialties was used as the cut-off for having a highly contested surgical resource. Resources for emergency surgery were assessed on a numeric rating scale (NRS) of 0–10, where 4 to 6 corresponded to having enough resources, 0–4 to having too few, and 6–10 to having an abundance. Cases were categorised as the speciality’s own case according to Table 1, “Speciality”.

### Statistical analysis

A complete case analysis was performed. No formal sample size calculation was undertaken, as the instrument had not previously been tested and the expected group sizes and variability were unknown. Respondent demographics were summarised using numbers (percentages) and medians with interquartile range (IQR), as appropriate. NRS responses were visualised in figures and compared across specialties. For each case, the median prioritisation score and IQR were calculated by speciality. Overall group differences between specialties were conducted using the Kruskal–Wallis test, followed, where appropriate, by Dunn’s post hoc test for pairwise comparisons. Cliffs Delta was used for effect sizes, this number can range from − 1 to 1 depending on the direction of differences [6, 7]. 

A logistic regression model was used to identify factors associated with daily prioritisation conflicts. The model included sex, speciality, clinical experience, perceived resources for emergency surgery,, highly contested surgical resource sharing(≥ 4 specialties sharing), the perception that there were routines for prioritisation (neutral, agree, and strongly agree vs. strongly disagree/disagree) and the perception that prioritisation impacts patient safety (neutral, agree, and strongly agree vs. strongly disagree/disagree). The variables were selected since they reflect demographics and the situation of the respondents, while questions about inter and intra-speciality relationships were considered potentially dependent on the rate of conflicts. Results were reported as odds ratios (OR) and 95% confidence intervals (CI).

Statistical analyses were conducted using R version 4.4.2 and R Studio 2024.09.1. P-values < 0.05 were considered statistically significant. The manuscript was prepared using the Consensus-Based Checklist for Reporting Of Survey Studies (CROSS) and applicable items from the Checklist for Reporting Results of Internet E-Surveys (CHERRIES) [8, 9]. 

## Results

Survey invitations were sent to 856 email addresses. Of the 315 survey responses initiated 204 (65%) were entered using a personal link (response rate of 204/856, 24%), and 156 of 204 (76%) used university-affiliated email addresses. The total response rate could not be calculated because the distribution also included posters and generic links; 111 of 315 (35%) responses came from posters or non-personal links. Out of 315 initiated responses 292 respondents completed the survey (93%). Of the 27 who did not complete the survey 15 were female, and 14 of 27 were registrars and there was an overrepresentation of anaesthetists, 10 of 27. Non-medical doctors (*n* = 24), and physicians not working with emergency surgery (*n* = 31) were excluded, leaving 233 respondents for analysis.

Among the 233 respondents, the majority were male (*n* = 137, 60%) and had over 15 years of clinical experience. Half (*n* = 129, 55%) reported sharing surgical resources with four or more other specialties. The most represented specialties were general surgery (*n* = 106, 45%), orthopaedic surgery (*n* = 36, 15%), and anaesthesiology (*n* = 34, 15%) (Table 2).

**Table 2:** Characteristics of responders working with emergency surgery to the survey on emergency surgery prioritisation

	Characteristic	*N* = 233
Sex	Female	91 (40%)
	Male	137 (60%)
	No reply	5
Experience	Registrar	52 (22%)
	Consultant	181 (70%)
Speciality	Anaesthesiology	34 (15%)
	General surgery	106 (45%)
	Neurosurgery	11 (4.7%)
	Obstetrics and gynaecology	19 (8.2%)
	Orthopaedic surgery	36 (15%)
	Urology	8 (3.4%)
	Vascular surgery	13 (5.6%)
	Other	6 (2.6%)
Length of experience (years)	<5	11 (4.7%)
	5–10	42 (18%)
	10–15	50 (21%)
	15–20	52 (22%)
	>20	78 (33%)
Shares emergency surgery resources	Yes	201 (90%)
Shared with ≥ 4 other specialties	Yes	129 (55%)

General surgery and obstetrics and gynaecology were most frequently identified as the most difficult specialties to agree with, selected by 45 and 47 of 233 respondents, respectively (25% and 26%). Anaesthetists and general surgeons most often cited obstetrics and gynaecology as challenging (32% and 35%, *p* = 0.03 and < 0.001), while obstetricians and gynaecologists and orthopaedic surgeons most frequently pointed to general surgery as difficult to agree with (62% and 54%, both *p* < 0.001).

### Resources and work environment

**Fig. 1:**
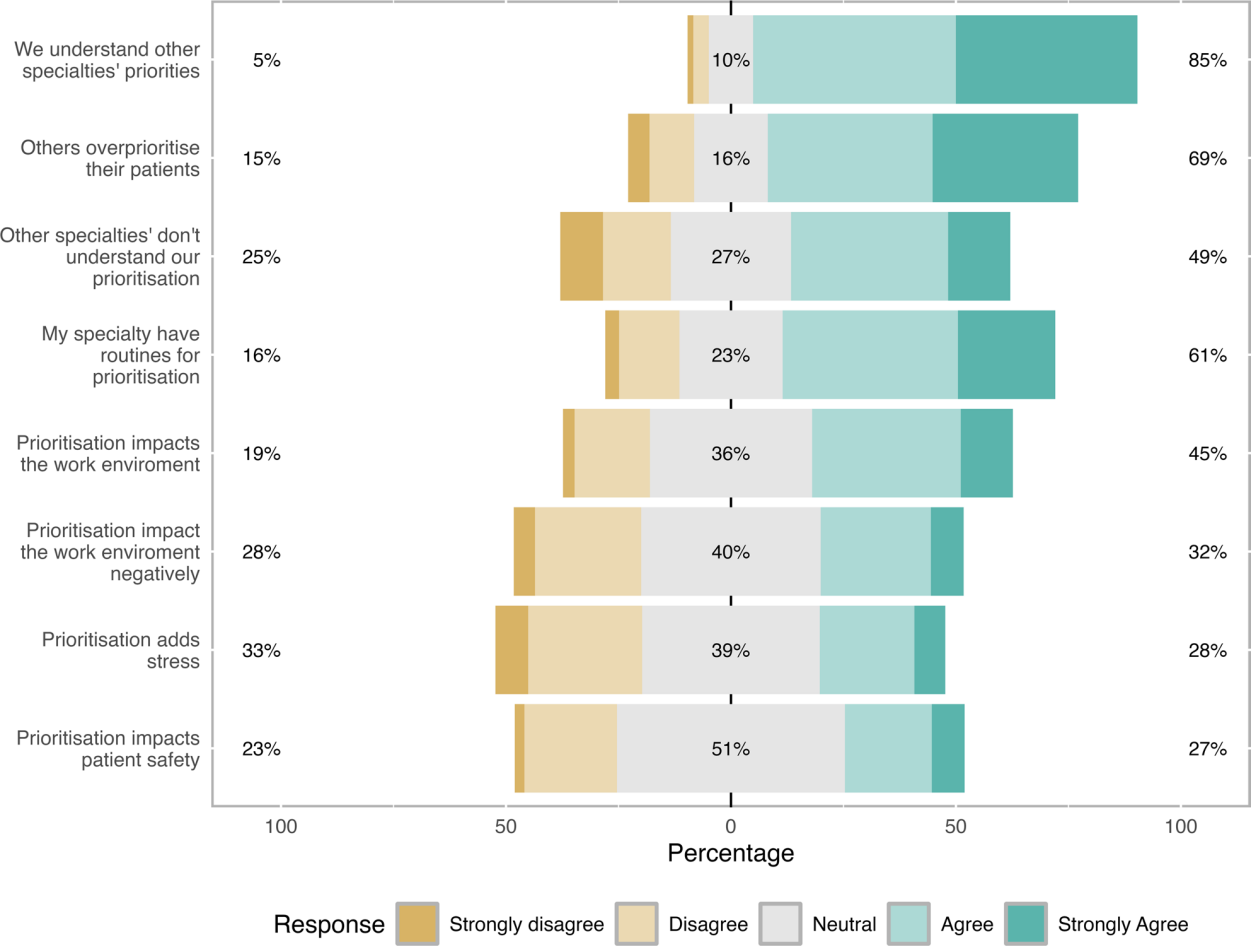
Perceptions of prioritisation and its impact among surgical specialties. Stacked bars show the distribution of responses to statements about routines, collaboration, and the perceived consequences of prioritisation. Values are expressed as percentages of responders selecting each option on a 5-point Likert scale ranging from *Strongly disagree* to *Strongly agree*

Most respondents felt that resources for emergency surgery were insufficient, median NRS score of 2.9 (IQR 2.1–3.8) on a 0–10 scale. One hundred fifty-six respondents (67%) gave scores below 4, and five (2%) above 6. Most (83%) believed their own speciality had internal consensus on prioritisation, but felt other specialties misunderstood them (49% agreed or strongly agreed vs. 25% disagreed or strongly disagreed) and over-prioritised their own patients (69%) (Fig. 1). While 61% reported having routines for prioritisation, opinions were divided on whether prioritisation impacted the work environment and patient safety. There were differences between specialties in perceived resources for emergency surgery (*p* = 0.02), self-assessed internal consensus (*p* = 0.03), use of routines for prioritisation (*p* = 0.01), perceived impact of prioritisation on the work environment (p = < 0.001), negative impact (*p* < 0.001), added stress (*p* = 0.01), and safety (*p* = 0.003). No differences between specialities regarding feeling misunderstood (*p* = 0.19) or perceiving over-prioritisation by other specialities (*p* = 0.06) were found.

Post hoc testing (Table 3) showed that anaesthetists perceived greater availability of resources for emergency surgery, stronger internal understanding within their own specialty, and more established routines for prioritisation. They also reported that prioritisation had a smaller impact on their work environment, stress, and patient safety than did many other specialties. General surgeons and obstetricians and gynaecologists considered prioritisation to have a smaller impact on their work environment and stress than several other specialties. In contrast, neurosurgeons reported that prioritisations had a greater and more negative impact on their work environment, that it added stress, and that it adversely affected patient safety.

**Table 3:** Pair-wise comparison of specialties responses on prioritisation routines and the impact on work environment, stress, and safety. The first specialty in each cell agreed more/less than the ones written below and indented. P-values and Cliffs Delta are in the same order

Statement	Agreed/happens more	*p*	CD	Agreed/happens less	*p*	CD
Du you have enough resources for emergency surgical procedures?	Anaesthetists than: general neurosurgeons orthopaedic vascular urologic OB/GYN than: orthopaedic	0.002 0.01 < 0.001 0.01 0.001 0.02	0.5 0.2 0.1 0.1 0.2 0.02			
We have a good understanding within my specialty of what others consider to be acute conditions.	Anaesthetists than: general neurosurgeons OB/GYN other	0.02 0.04 0.003 0.007	0.3 0.3 0.4 0.6			
We have procedures at my workplace for how surgical cases should be prioritised.	Anaesthetists than: neuro- orthopaedic vascular	0.03 0.04 < 0.001	0.3 0.2 0.6	Vascular than: general OB/GYN	< 0.001 0.002	0.5 0.5
Does prioritisation affect your work environment?	Neurosurgeons than: Anaesthetists general OB/GYN orthopaedic urologic vascular	0.009 < 0.001 < 0.001 0.007 0.02 0.01	0.5 0.7 0.5 0.5 0.6 0.5	General than: Anaesthetists orthopaedic other	0.01 0.01 0.001	0.3 0.3 0.7
Does prioritisation negatively affect you?	Neurosurgeons than: General	< 0.001	0.6	OB/GYN than: neurosurgeons- orthopaedic vascular other Anaesthetists than: neurosurgeons- orthopaedic vascular other	< 0.001 0.009 0.03 0.006 < 0.001 0.005 0.03 0.006	0.7 0.4 0.4 0.7 0.7 0.3 0.3 0.6
Does prioritisation affect your stress level?	Neurosurgeons than: Anaesthetists general OB/GYN orthopaedic urologic vascular	0.002 < 0.001 0.001 0.03 0.02 0.02	0.6 0.5 0.6 0.3 0.7 0.5	General and OB/GYN than: other	0.01 0.01	0.1 0.5
Does prioritisation negatively affect patient safety?	Neurosurgeons than: general OB/GYN orthopaedic urologic	< 0.001 < 0.001 0.01 0.01	0.5 0.6 0.4 0.6	Anaesthetists than: Neurosurgeons vascular other OB/GYN than: Orthopaedic vascular other	< 0.001 0.03 0.01 0.04 0.01 0.007	0.6 0.3 0.6 0.3 0.4 0.6

*OB/GYN* Obstetrics and gynaecology, *CD* Cliffs Delta, a measure of effect sizes. Here the effect size is indicated by the column. Small: 0.1–0.3, Medium:0.3–0.4, Large > 0.4

## .Prioritisation of cases

**Fig. 2:**
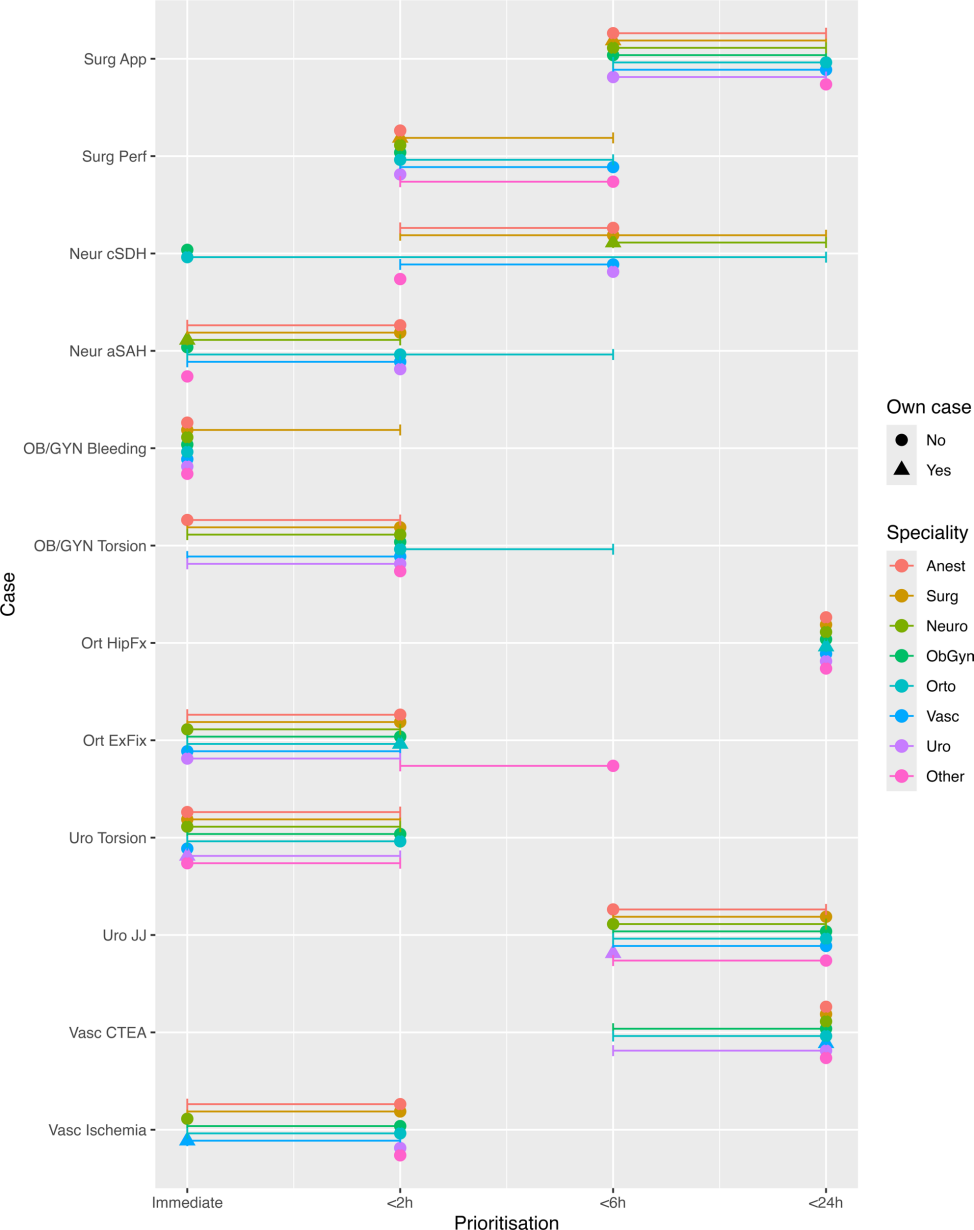
Variation in prioritisation of emergency surgical cases across specialties. Each circle or triangle represents the median prioritisation (Immediate (0), 2, 6 and 24 h) for a given speciality and case, with horizontal bars denoting the interquartile range. Triangles indicate prioritisation of the specialties’ own cases

**Fig. 3:**
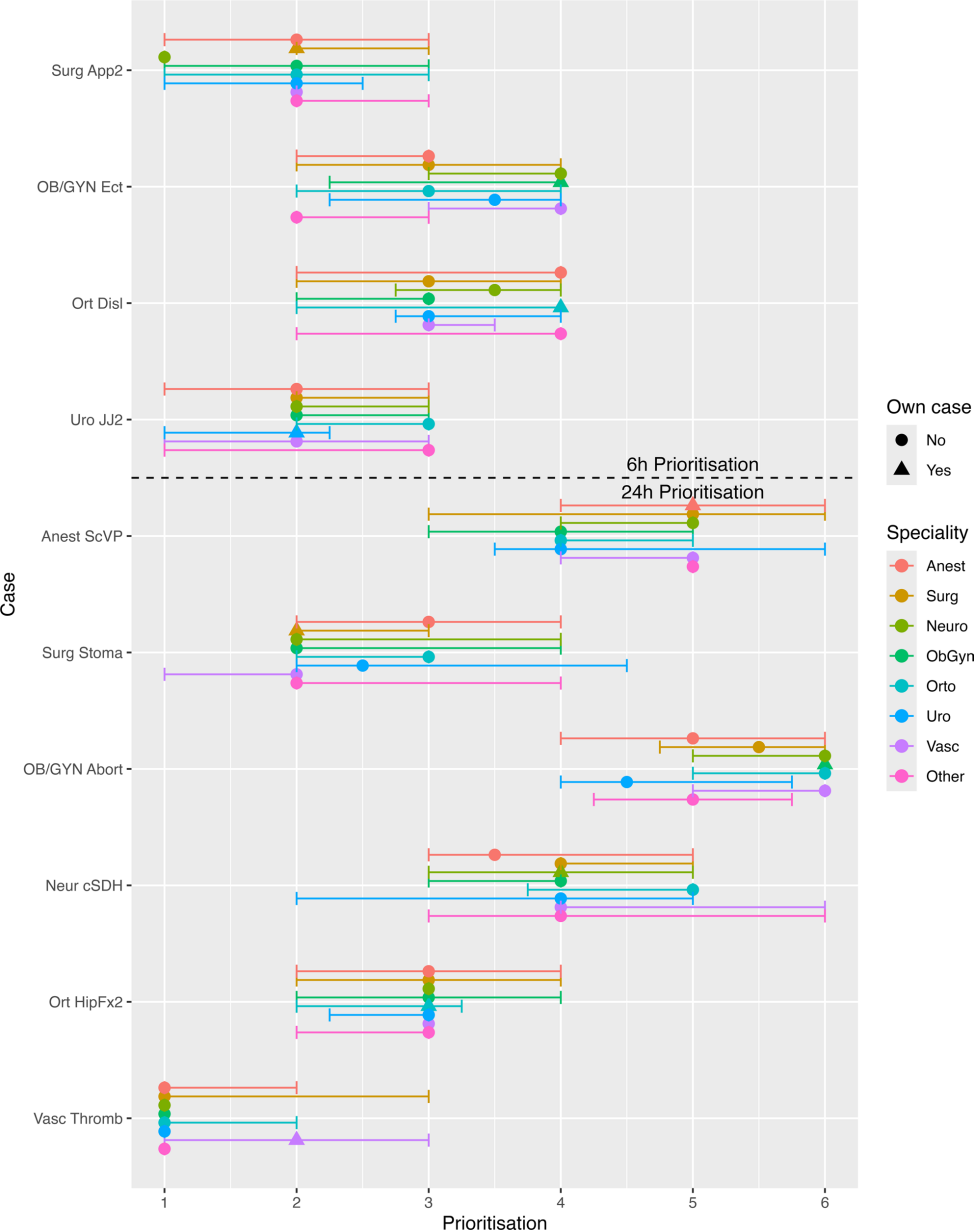
Variation in prioritisation of within 6- and 24-hour emergency surgical cases across specialties. Respondents ranked which cases should be performed first among those already classified as requiring surgery within 6–24 h. Each circle represents the median prioritisation rating for a given speciality and case, with horizontal bars denoting the interquartile range. Triangles indicate prioritisation of the specialties’ own case. Dashed lines separate the 6-hour and 24-hour prioritisation groups

Differences in prioritisation between specialties were observed for a limited number of hypothetical cases. Among the immediate/<2 h/<6 h/<24 h cases, three had differences between specialties: OB/GYN torsion, urology JJ, and vascular CTEA (all *p* ≤ 0.01; Fig. 2; Table 4). Anaesthetists prioritised the OB/GYN torsion case higher than other specialties, while orthopaedic surgeons assigned a lower priority to it than general, vascular, and urologic surgeons. For the urology JJ case, anaesthetists assigned a higher priority than obstetricians and gynaecologists, while orthopaedic surgeons assigned a lower priority than anaesthetists, general, and vascular surgeons. For vascular CTEA, anaesthetists prioritised this case lower than orthopaedic and urologic surgeons, while obstetricians and gynaecologists assigned a higher priority to the Vascular CTEA case than other specialties. For prioritisation between cases scheduled within six hours (Fig. 3), there were differences between specialties for appendectomy (*p* = 0.03). General surgeons prioritised this case lower than orthopaedic surgeons, while neurosurgeons prioritised it higher than other specialties. For cases scheduled within 24 h (Fig. 3), there were differences for the general surgery stoma case (*p* = 0.01), OB/GYN Abortion (*p* = 0.04), and Vascular Thrombolysis (*p* = 0.005). Post hoc testing is presented in Table 4. For the general surgery stoma case, anaesthetists prioritised it lower than general and vascular surgeons. Obstetricians and gynaecologists assigned a lower priority to the OB/GYN Abortion case than other specialties. For the Vascular Thrombolysis case, general and vascular surgeons assigned a lower priority than other specialties.

**Table 4:** Pair-wise comparisons of case prioritisation for cases where the overall test for differences indicated a difference. The first section of the table details differences between specialties and time-based prioritisation of cases where respondents could prioritise cases as immediate, < 2, <6 and < 24 h. The second section details the differences where cases were ranked. The first specialty in each cell prioritised higher/lower than the ones written below and indented. P-values and Cliffs Delta are in the same order

Immediate/<2 h/<6 h/<24 h Prioritisation	Prioritised higher	*p*	CD	Prioritised lower	*p*	CD
OB/GYN Torsion	Anaesthetists than: general, OB/GYN, orthopaedic, other	0.01 0.02 <0.001 0.009	0.1 0.5 0.4 0.6	Orthopaedic than: general, vascular, urologic	0.002 0.01 0.03	0.4 0.4 0.3
Urology JJ	Anaesthetists than: OB/GYN, orthopaedic	0.02 <0.001	0.5 0.5	Orthopaedic than: general, vascular	0.01 0.004	0.3 0.1
Vascular CTEA	OB/GYN than: general, neurosurgeons, vascular, other	0.001 0.003 0.03 0.02	0.1 0.5 0.4 0.6	Anaesthetists than: OB/GYN, orthopaedic, urologic	< 0.001 <0.001 0.01	0.2 0.2 0.3

**Ranked cases**	**Prioritised higher**	**p**	**CD**	**Prioritised lower**	**p**	**CD**
General Appendicitis	Neurosurgeons than: anaesthetists, OB/GYN, orthopaedic, urologic, vascular	0.003 0.01 0.01 0.04 0.02	0.3 0.4 0.3 0.3 0.3	General than: Neurosurgeons, orthopaedic	< 0.001 0.02	0.3 0.3
General Stoma	Vascular than: general, neurosurgeons, OB/GYN, orthopaedic, urologic	0.01 0.01 0.02 0.003 0.03	0.3 0.7 0.3 0.6 0.6	Anaesthetists than: general, vascular	0.002 < 0.001	0.4 0.6
OB/GYN Abortion	Anaesthetists than: OB/GYN, orthopaedic	< 0.001 0.02	0.6 0.4	OB/GYN than: general, urologic, other	0.004 0.009 0.01	0.4 0.1 0.4
Vascular Thrombolysis				General than: anaesthetists, neurosurgeons, OB/GYN, orthopaedic Vascular than: anaesthetists, neurosurgeons, OB/GYN, orthopaedic, urologic, other	0.05 0.006 0.002 0.04 0.008 0.001 < 0.001 0.007 0.03 0.05	0.01 0.1 0.1 0.03 0.4 0.3 0.6 0.3 0.04 0.2

*CD* Cliffs Delta, a measure of effect sizes. Here the effect size is indicated by the column. Small: 0.1–0.3, Medium:0.3–0.4, Large > 0.4

### Factors impacting conflicts

Prioritisation conflicts were common: 39 respondents (17%) reported daily conflicts, whereas 18 (8%) reported never experiencing such conflicts. Multivariable logistic regression of variables likely associated with conflicts (Table 5) showed that increasing concerns about compromised patient safety (OR 8.05 and 26.9 for those agreeing and strongly agreeing vs. strongly disagreeing/disagreeing, 95% CI 1.60–63.5 and 4.03–263, respectively) and increasing resources (OR 0.68 95% CI 0.48–0.96) were associated with daily conflicts.

**Table 5:** Logistic regression for daily or more than daily conflict regarding prioritisation of cases

Characteristic	OR	95% CI
*Male vs. Female*	1.04	0.36–3.14
*Speciality (Reference General Surgery)*		
* Anaesthesia*	3.37	0.87–13.0
* Obstetrics and gynaecology*	3.2	0.49–18.3
* Orthopaedics*	1.54	0.37–5.96
* Other*	0.88	0.24–2.99
*Length of healthcare experience (years)*		
* 5–10 vs. < 5*	2.58	0.23–68.6
* 10–15 vs. < 5*	0.41	0.02–12.5
* 15–20 vs. < 5*	2.01	0.17–54.7
* >20 vs. < 5*	3.23	0.31–82.9
*Perceived resources for emergency surgery (1–10*,* Too few – enough – more than enough)*	0.68	0.48–0.96
*Share resources with ≥ 4 specialties*,* Yes vs. No*	1.13	0.44–3.03
*We have routines for prioritisation*		
* Neutral vs. Strongly Disagree/Disagree*	0.26	0.05–1.10
* Agree vs. Strongly Disagree/Disagree*	0.41	0.11–1.51
* Strongly Agree vs. Strongly Disagree/Disagree*	0.69	0.17–2.79
*Prioritisation impacts safety*		
* Neutral vs. Strongly Disagree/Disagree*	2.8	0.65–19.6
* Agree vs. Strongly Disagree/Disagree*	8.05	1.60–63.5
* Strongly Agree vs. Strongly Disagree/Disagree*	26.9	4.03–263

*95%CI* 95% Confidence Interval, *OR* Odds Ratio

## Discussion

In this survey of physicians involved in emergency surgery, participants frequently perceived that other specialties did not understand their prioritisation criteria and tended to over-prioritise their own cases. However, when asked to prioritise hypothetical cases, we did not observe any scenario in which the specialty that usually manages the condition prioritised their “own” case higher than other specialties. Contrary to our hypothesis, when faced with several urgent cases, physicians tended to prioritise cases from their own specialty lower than those from other specialties. The findings suggest that perceived inter-specialty differences are greater than the actual differences observed when prioritising hypothetical cases.

Concerns about patient safety were associated with perceived prioritisation conflicts, potentially reflecting physicians’ sense of responsibility for ensuring timely surgery for their patients. In contrast, greater perceived availability of resources for emergency surgery appeared to reduce the perceived need to compete for operating resources and may facilitate prioritisation based on clinical urgency. General surgery and obstetrics and gynaecology, two specialties with a high volume of emergency cases, were most frequently perceived as difficult to collaborate with on prioritisation. These specialties also perceived the negative impact of prioritisation as smaller than did many other specialties, which may indirectly suggest that they more often proceed ahead of others. In contrast, neurosurgeons perceived that prioritisation had a more negative impact, adding stress and adversely affecting patient safety to a greater extent than in other specialties. This could reflect a high number of emergency cases or stronger competition for limited resources in neurosurgery, although our data cannot determine the underlying cause.

Other studies have examined inter-speciality concordance in the American Society of Anesthesiologists (ASA) classification between anaesthetists and other specialties, demonstrating moderate agreement in real-world settings, but with other specialties often underrating patients in hypothetical cases [10–12]. Studies have also analysed which case codes are booked as urgent in routine medical records [13] and whether survival is associated with assigning the correct priority to the case [14]. However, the prioritisation process, and its impact on workplace dynamics and the individual, is less well studied. Organisational factors may also shape behaviour: cases that do not medically require surgery during on-call hours may nonetheless be performed at night to avoid disrupting elective lists, expedite discharge, or enable repatriation to other hospitals. A clearer understanding of how physicians across specialties perceive urgency and negotiate shared operating resources could inform structured, transparent prioritisation frameworks. Such initiatives may reduce perceived inequities and improve interprofessional collaboration in emergency surgery.

### Strengths and weaknesses

To our knowledge, this is the first study investigating how physicians prioritise emergency surgical cases across specialties. The study provides an initial framework for further research by defining areas of disagreement and organisational influences. The sample represents a subset of Swedish physicians involved in emergency surgery with an over-representation of university hospitals, as email addresses for the initial invitations came from university web pages and can not be considered random. Further information on respondents, such as hospital size and setting, would aid in interpreting external validity. In total, approximately 315 responses were initiated; 233 responses were complete and eligible (physicians working with emergency surgery). Respondents who were younger career-wise were less likely to respond to the survey. These physicians are usually less involved in the prioritisation of cases and resources; however, this decreases the generalisability of the results. There was an English version of the survey, but international participation among respondents is unlikely. Social desirability bias is possible, as respondents may have avoided ranking other specialties’ cases unrealistically low or their own cases unrealistically high [15]. No control for multiple participation was performed.

A follow-up study with international distribution and a parallel survey of medical students is planned to compare systems, assess knowledge of surgical prioritisation, and explore whether prioritisation is explicitly taught during training.

## Conclusion

Most physicians agreed that resources for emergency surgery are insufficient. Insufficient resources and concerns about patient safety were associated with daily conflicts concerning prioritisation. Although many perceived that other specialties did not understand their prioritisation, prioritisation of hypothetical cases differed little between specialties. Further studies should include international cohorts and examine organisational factors contributing to prioritisation dynamics.

## Supplementary Information

Supplementary Material 1.

Supplementary Material 2.

## Data Availability

The datasets generated and analysed during the current study are available from the corresponding author on reasonable request.

## Abbreviations

95% CI: 95% Confidence Interval
ASA: American Society of Anesthesiologists
CRP: C-reactive Protein
GCS: Glasgow Coma Scale
OB/GYN: Obstetrics and Gynaecology
OR: Odds Ratio
p: p-value
RLS: Reaction Level Scale
