# Supplementary material for: Surveying prioritisation for emergency surgery - do the specialties involved agree?
Source: BMC Surg. 2026 Jan 28;26:153. doi: 10.1186/s12893-026-03538-3 (PMC12922434; doi:10.1186/s12893-026-03538-3)
Supplement: Supplementary file 2 — Supplementary Material 2. [file 12893_2026_3538_MOESM2_ESM.pdf]

Survey on emergency surgery prioritisation

The aim is to study how different specialties and professional groups prioritize patients from different areas and whether this affects conflicts around this.

This is a survey on emergency surgery prioritisation.

You will first be asked about your workplace, if you are involved in emergency surgery and conflicts around prioritisation. The purpose of this survey is to study how different specialties prioritise their and others cases. The survey takes approximately 5 minutes to complete. Your responses can not be linked to you.

GDPR

Your personal information (email adress) will be treated in accordance with GDPR. The legal ground for data processing is necessary for the performance of a task carried out in the public interest (research). Your personal data is encrypted and protected by 2-factor authentication. This will be stored separatly from your survey answers. You have the right to request information on the information stored, have them corrected, deleted and object to their handling. The answeres will be archived in accordance with Swedish Law. Your email will be deleted once the survey closes. If you have questions on the handling of your personal data please contact the data protection officer at Uppsala University, dataskyddsombud@uu.se

The responsible researcher is

Erik Osterman
Uppsala Universitet, Institutionen för kirurgiska vetenskaper
Akademiska Sjukhuset, Ing 70 751 85, Uppsala, Sverige
emsurg.prio.surv@gmail.com

Background information

Do you work with emergency surgeries?

- ◯ Yes
- ◯ No

What's your title/job?

- ◯ Resident
- ◯ Fellow/Attending
- ◯ Nurse
- ◯ Anesthesia Nurse/Anesthesia Advanced practice provider
- ◯ Scrub nurse /Surgical tech
- ◯ Circulating nurse or tech
- ◯ Student
- ◯ Other

What specialty do you practice within?

- ☐ Antesthesia
- ☐ Surgery
- ☐ Vascular Surgery
- ☐ Neurosurgery
- ☐ Obstetrics/Gynecology
- ☐ Orthopedics
- ☐ Urology
- ☐ Other

Enter other specialty?

____

How long have you worked within health care?	◯ < 5 years<br>◯ 5-10 years<br>◯ 10-15 years<br>◯ 15-20 years<br>◯ >20 years
In which country do you work?	◯ Africa<br>◯ Asia<br>◯ Europe<br>◯ North America<br>◯ Oceania<br>◯ South America
What is your sex?	◯ Female<br>◯ Male<br>◯ Other/Don't want to say

Surgery prioritisation at work

Do you share resources with another specialty for emergency surgical procedures?

- ◯ Yes
- ◯ No

Resources: Operating room, scrub nurse/techs, anesthesia resources (i.e. the things needed to make emergency procedures happen)

Are you involved in the prioritisation of emergency surgery? (e.g. on call, planner etc.)

- ◯ Yes
- ◯ No

Which specialties?

- ☐ Anesthesia
- ☐ Surgery
- ☐ Vascular Surgery
- ☐ Neurosurgery
- ☐ Obstetrics/Gynecology
- ☐ Orthopedics
- ☐ Urology
- ☐ Other

What other?

____

Du you have enough resources for emergency surgical procedures?

Not at all — Enough — To much

(Place a mark on the scale above)

How often are there conflicts about prioritisation of emergency surgical procedures in your work place?

- ◯ Never
- ◯ Once a month
- ◯ Once a week
- ◯ Once per day
- ◯ Multiple times per day

Which specialty is it the hardest to agree with on?

- ◯ Anesthesia
- ◯ Surgery
- ◯ Vascular surgery
- ◯ Neurosurgery
- ◯ Obstetrics/Gynecology
- ◯ Orthopedics
- ◯ Urology
- ◯ Other

What other?

____

To what extent do you agree with the following statements?

	Completely disagree	Partially disagree	Neutral	Partially agree	Completely Agree
We have a good understanding within my specialty of what others consider to be acute conditions.	○	○	○	○	○
I feel that some specialties over-prioritise their patients at the expense of others.	○	○	○	○	○
I feel that other specialties underestimate how urgent the conditions we handle are.	○	○	○	○	○
We have procedures at my workplace for how surgical cases should be prioritised.	○	○	○	○	○

Impact of emergency surgery prioritisation

	Never	Seldom	Sometimes	Often	Almost always
Does prioritisation affect your work environment?	○	○	○	○	○
Does prioritisation negatively affect you?	○	○	○	○	○
Does prioritisation affect your stress level?	○	○	○	○	○
Does prioritisation negatively affect patient safety?	○	○	○	○	○

Emergency cases, information

You will now be given several fictional cases who need emergency surgery.

Each case is a few senteces describing the situation. Please prioritise these cases on how fast the intervention needs to happen.

Immediate - Patient needs surgery now. No time to optimise, preoperative shower etc. It should already be here.

Within 2 hours - If the OR is occupied, the current surgery can finish but it's importat that the patient has surgery soon.

Within 6 hours - Can not wait until day if it's now night.

Within 24 hours - Can wait until it's day.

Prioritisation of cases

	Immidiate	< 2 hours	< 6 hours	< 24 hours
Laparoscopic appendectomy. 13-year old boy with appendicitis. CRP 50. Local peritonitis. No fever. Not septic.	○	○	○	○
Laparotomy. 56 year old man with diverticulitis. CT with free fluid and some gas. Septic with peritonitis.	○	○	○	○
Sliding hip screw and plate. 85-year-old woman. A stroke 2 months ago. Pertrochanteric femur fracture after fall.	○	○	○	○
Debridement and external fixation. 25-year-old woman with open tibia fracture after motocross accident. Impaired circulation in the foot.	○	○	○	○
Exploration of testicle. 8-year-old with suspected testicular torsion. Pain since 4 hours. Otherwise well.	○	○	○	○
Double J-stent. 55-year-old man with urinary tract stones. Fever. No sepsis.	○	○	○	○
Removal of retained placenta. 32-year-old woman with postpartum hemorrhage. So far 1500 ml of bleeding.	○	○	○	○
Laparoscopy. 17-year-old with suspected ovarian torsion. Severe pain. Otherwise well.	○	○	○	○
Carotid endarterectomy. 76-year-old woman with a stroke 2 days ago. 90% stenosis of left carotid.	○	○	○	○
Thromboembolectomy lower extremity. 60-year-old man with atrial fibrillation. Cold, pulseless leg with loss of sensation.	○	○	○	○

Haemorrhage evacuation. 78-year-old man, prostate cancer, hypertension and alcohol abuse. Increasing hemiparesis, very weak against gravity. CT with an expansive chronic subdural hematoma.	○	○	○	○
Ventricular drainage. 54-year-old woman, smoker, hypertension. Subarachnoid hemorrhage from common artery aneurysm. Declining level of consciousness RLS1/GCS 15 to RLS3a / GCS 11. Progression of hydrocephalus on control CT.	○	○	○	○

All these surgeries are prioritised for within 6 hours. In which order do you think they should be performed?

	1	2	3	4
Laparoscopy. 25-year-old woman with ectopic pregnancy, not ruptured. Stable blood pressure.	○	○	○	○
Laparoscopic appendectomy. 17-year-old woman with appendicitis. Fever. Peritonitis.	○	○	○	○
Reduction under general anesthesia. 24-year-old man with elbow dislocation.	○	○	○	○
Double J-stent. 55-year-old woman with suspected blocked pyelitis due to ureteral stone.	○	○	○	○

All these surgeries are prioritised for within 24 hours. In which order do you think they should be performed?

	1	2	3	4	5	6
Placement of a transversostomy. 50-year-old man. Obstructing rectal cancer.	○	○	○	○	○	○
Hemiprosthesis. 85-year-old woman. Hip fracture.	○	○	○	○	○	○
Surgical abortion. 39-year-old woman. Intrauterine pregnancy without bleeding.	○	○	○	○	○	○
Subcutaneous venous port. 18-year-old male. Leukemia.	○	○	○	○	○	○
Thrombolysis. 73-year-old man. Critical ischemia with pain and ulceration in the right leg.	○	○	○	○	○	○
Evacuation of chronic subdural hematoma. 71-year-old woman. Moderate bilateral subdural hematomas. Head injury 6 weeks ago, mild headache and balance problems.	○	○	○	○	○	○
